# Single Nucleotide Polymorphisms in Pediatric Idiopathic Nephrotic Syndrome

**DOI:** 10.1155/2016/1417456

**Published:** 2016-05-09

**Authors:** Maija Suvanto, Timo Jahnukainen, Marjo Kestilä, Hannu Jalanko

**Affiliations:** ^1^Children's Hospital, University of Helsinki and Helsinki University Hospital, 00290 Helsinki, Finland; ^2^Department of Chronic Disease Prevention, National Institute for Health and Welfare, 00271 Helsinki, Finland

## Abstract

Polymorphic variants in several molecules involved in the glomerular function and drug metabolism have been implicated in the pathophysiology of pediatric idiopathic nephrotic syndrome (INS), but the results remain inconsistent. We analyzed the association of eleven allelic variants in eight genes* (angiopoietin-like 4 (ANGPTL4), glypican 5 (GPC5), interleukin-13 (IL-13), macrophage migration inhibitory factor (MIF), neural nitric oxide synthetase (nNOS), multidrug resistance-1 (MDR1), glucocorticoid-induced transcript-1 (GLCCI1),* and* nuclear receptor subfamily-3 (NR3C1))* in 100 INS patients followed up till adulthood. We genotyped variants using PCR and direct sequencing and evaluated estimated haplotypes of MDR1 variants. The analysis revealed few differences in SNP genotype frequencies between patients and controls, or in clinical parameters among the patients. Genotype distribution of MDR1 SNPs rs1236, rs2677, and rs3435 showed significant (*p* < 0.05) association with different medication regimes (glucocorticoids only versus glucocorticoids plus additional immunosuppressives). Some marginal association was detected between* ANGPTL4*,* GPC5*,* GLCCI1,* and* NR3C1* variants and different medication regimes, number of relapses, and age of onset.* Conclusion.* While* MDR1* variant genotype distribution associated with different medication regimes, the other analyzed gene variants showed only little or marginal clinical relevance in INS.

## 1. Introduction

Childhood-onset idiopathic nephrotic syndrome (INS) is a common kidney disease in children. It is characterized by minimal glomerular changes in light microscopy and podocyte foot process effacement in electron microscopy. The great majority (80–90%) of INS patients show good responsiveness to steroid treatment, but recurrent episodes occur in at least 70% of the patients. Development of renal failure occurs rarely.

The pathophysiology of INS is still unknown. During the past few years, polymorphic variants of several molecules involved in the glomerular function have been analyzed in INS patients and animal models of proteinuria to discover genetic components that would participate in the development of disease or modify its phenotype. These include angiopoietin-like 4 (*Angptl4*), glycoprotein, which is upregulated in rats with steroid sensitive proteinuria [1, 2], interleukin-13 (*IL-13*), a cytokine that alters podocyte function and is reported to be upregulated in patients with active INS [3, 4], macrophage migration inhibitory factor (*MIF*), a proinflammatory cytokine and counter regulator of the immunosuppressive effects of glucocorticoids [5, 6], glypican-5 gene (*GPC5*), which encodes a podocyte cell surface proteoglycan whose variants are associated with a podocyte injury and proteinuria [7], and neural nitric oxide synthetase (*nNOS*) that plays a role in glomerular hyperpermeability [8].

In addition to the five functional molecules, there are reports on the association of INS with polymorphic variants of molecules involved in the glucocorticoid metabolism. These include multidrug resistance-1 gene (*MDR1*) encoding P-glycoprotein-170 (P-gp), which transports steroids across the cell membranes [9–12], nuclear receptor subfamily-3 gene (*NR3C1*) that encodes cytosolic glucocorticoid receptor (GR) [13, 14], and glucocorticoid-induced transcript 1 gene (*GLCCI1*) that encodes Glcci1 protein that associated with glucocorticoid (GC) responsiveness and development of proteinuria in animal models [15, 16].

In this study, we genotyped eleven single nucleotide polymorphisms (SNPs) from the above mentioned eight genes from DNA samples obtained from a unique cohort of INS patients followed up over 30 years. The results show that the variants in the genes coding for functional kidney proteins have little clinical relevance in INS while variants in genes involved in glucocorticoid metabolism show marginal association with INS. The genotype distribution of MDR1 SNPs shows significant association with medication regime as the minor alleles of the SNPs are more frequent in patients who receive immunosuppressive medication in addition to glucocorticoids.

## 2. Materials and Methods

### 2.1. Patients

The study cohort included 100 INS patients of whom 83 were diagnosed within 1965–1981 at the Hospital for Children and Adolescents, University of Helsinki. These 83 patients were enrolled in the International Study of Kidney Disease in Children (ISKDC) and were treated by protocols still used in pediatric nephrology. The mean age at the last follow-up was 35.0 years (range 25.1–44.1 years). Clinical data were carefully recorded from the patient records as was previously reported in detail [17]. The remaining 17 patients were diagnosed more recently at the Hospital for Children and Adolescents, University of Helsinki, and were still children or adolescents; of them SNP genotypes were used in statistical comparison between INS patients and controls. A blood sample for DNA extraction was gathered from all.

The controls included a cohort of 101 subjects who were either healthy adults or suffered from nonkidney related condition.

### 2.2. SNPs and PCR Analysis

We selected eleven SNPs from eight genes to this study based on previous reports in literature on their association with INS. The genes and SNPs were* Angptl4* SNP rs1044250 (c.797C>T),* GPC5* SNP rs16946160 (c.325+102637G>A),* IL-13* SNP rs848 (c.^*∗*^526C>A),* MIF* rs755622 (c.-270G>C),* nNOS* rs2682826 (c.^*∗*^276C>T),* MDR1* SNPs rs1128503 (c.1236C>T), rs2032582 (c.2677G>T/A), rs1045642 (c.3435C>T),* GLCC11* SNPs rs37972 (c.-1473C>T), and rs37973 (c.-1106A>G) and* NR3C1* rs41423247 (c.1184+646G>C). Analysis was carried out using direct sequencing. Exons were amplified by PCR with flanking intronic primers, and the reactions were performed in total volumes of 25 *μ*L as previously described [18]. Occasionally, the denaturation temperature was raised up to 98°C, or betaine was added to the reaction mixture. PCR products were genotyped using automated sequence analysis by BigDye-terminator chemistry (v.3.1) on Genetic Analyzer 3730 (Applied Biosystems). The sequences were analyzed with GeneComposer version 1.1.0.1051 (http://www.GeneComposer.com/). The genotypes were compared between patients and controls and among various clinical variables that include age of onset (<3 yr versus >3 yr), number of relapses (<5 versus >5), frequent relapses (no versus yes), response to GC (normal versus slow/no response), treatment (only GC versus GC together with immunosuppressive (IS) drugs), and GC dependence (no versus yes).

The genotypes of the three* MDR1* variants (rs1236, rs2677, and rs3435) were used to carry out a haplotype analysis. Twelve haplotypes were derived from the genotype data using the program PHASE (v2.1) [19, 20]. The estimated* MDR1* haplotype frequencies were compared between patients and controls and among various clinical variables.

All primers are presented in Table 1. Most of the primer sequences are gathered from literature:* Angptl4* rs1044250 [21],* IL-13* rs848 [4],* MIF* rs755622 [22],* nNOS* rs2682826 [8],* MDR1* rs1128503, rs2032582, rs1045642 [9], and* NR3C1* rs41423247 [23]. Primers for* GLCC11* (NM_138426), rs37972 and rs37973, and* GPC5* (NM_004466) rs16946160 were designed using primer3 program (http://www.ncbi.nlm.nih.gov/tools/primer-blast/).

### 2.3. Statistics

The statistical analysis was carried out using statistical software JMP 10 (http://www.jmp.com/, SAS institute Inc., Cary, NC, USA). Logistic regression analysis was used to calculate odds ratios (OD) and 95% confidence intervals (CI) for the association between genotypes and the risk of INS. The difference between selected clinical variables and genotypes was determined by Fisher's exact test. Statistical significance was defined as *p* < 0.05.

### 2.4. Ethics

All patients or their parents were informed of the content of the study and they signed a form of consent. The Ethics Committee of the Children's Hospital, University of Helsinki, approved the study (IRB: HUS509/E7/05).

## 3. Results

Eleven SNPs from eight genes encoding proteins related to kidney function and steroid metabolism (*Angptl4*,* GPC5*,* MIF*,* nNOS*,* IL-13*,* MDR1,* and* NR3C1 GLCCl1*) were analyzed from 100 INS patients. The distribution of observed genotypes was consistent with those expected under the assumptions of the Hardy-Weinberg Equilibrium (*p* ≥ 0.05) (Table 2).

Comparison of the frequencies of the 11 SNPs between INS patients and controls was made and is presented in Table 3. No association between variants and disease status was observed except in rs848 variant in* IL-13* gene, where heterozygotic genotype showed difference in frequency between patients and controls (53% versus 38%, OR 2.025, CI 1.095–3.785, and *p* = 0.0243).

Comparison of the SNP genotype frequencies among INS patients with various clinical parameters is presented in Tables 4 and 5. These variables included age of onset (<3 yr versus >3 yr), number of relapses (<5 versus >5), frequent relapses (no versus yes), response to GC (normal versus slow/no response), treatment (only GC versus GC together with immunosuppressive (IS) drugs), and GC dependence (no versus yes).

Few clinically relevant correlations were found in relation to the SNPs in genes* IL13*,* MIF,* and* nNOS*.* Angptl4* SNP rs1044250 allele C was more frequent in patients who received IS medication in addition to GCs compared to those who only received GCs (72 versus 56%, *p* = 0.0228). In* GPC5*, SNP rs16946160 A allele was more frequent in patients with disease onset less than three years of age than in those with disease onset more than three years of age (16 versus 5%, *p* = 0.0421).

The genotype distribution in* MDR1* SNPs showed difference in patients who received only GCs compared to those who also received IS medication as allele T frequency was higher in the latter group in rs1128503 (70.6 versus 28.6%, *p* = 0.0012), in rs2032582 (67.9 versus 42.9%, *p* = 0.0028) and in rs1045642 (73.1 versus, 52.4%, *p* = 0.0092). Similarly, in* GLCCI1* SNP rs37973 A allele was more frequent in patients who received IS medication (67 versus 50%, *p* = 0.0387). Patients with more than five relapses also had* GLCCI1* SNP rs37973 A allele more frequently than those with fewer relapses (70 versus 52%, *p* = 0.0377). A curious finding was that* NR3C1* SNP rs41423247 heterozygous GC genotype was more frequent in patients with more than five relapses (68 versus 32.7%) and in patients with frequent relapses (60 versus 34%).

Haplotype analysis of the three MDR1 variants (rs1236, rs2677, and rs3435) revealed twelve estimated haplotypes among cases and controls. These data are presented in Table 6. Curiously, the patient samples were less varied, as the allele frequency of the two most common haplotypes, TTT and CGC, was 70% (42.2 and 27.5%, resp.) while in the control samples it took the four most common haplotypes, TTT, CGC, CTT, and TGT, to achieve the same (23.4, 18.6, 15.6 and 12.6%, resp.). Most of the haplotypes had *p* < 0.05. The distribution of the allele frequencies of the haplotypes among clinical variables showed no significant difference between groups with the exception of the comparison between patients with less than five relapses and patients with more than five relapses, as is seen in Table 6. In other variants, only TTT and CGC haplotypes had allele frequency higher than 10% in any of the variables and the combined allele frequency of these two varied between 71 and 86.7%.

## 4. Discussion

The discovery of causative or disease modifying genetic factors underlying INS is of great interest and could have a profound clinical impact. In this study, we genotyped eleven SNPs from eight genes that had previously been studied in relation to INS and proteinuric animal models. We compared the SNP frequencies in INS patients and controls as well as in the subgroups of INS patients. Very little alteration was detected in the distribution of SNPs between patients and controls, and only marginal differences were observed among the INS subgroups in the* Angptl4*,* GPC5*,* MDR1,* and* NR3C1* genes. An exception is the genotype distribution of* MDR1* SNPs in patients who receive GC medication and those who also receive IS as the T alleles are more frequent in the latter group. Summation of the findings of this study and a comparison to other recent studies can be found in Table 7.

*Angptl4* is a secretory protein involved in lipid metabolism and its increased expression has been observed in podocytes and circulation in human and experimental INS [24, 25]. The genetic variant SNP rs1044250 in exon 6 leads to amino acid change p.T266M and the homozygous C genotype of this variant has been associated with lower plasma* Angptl4* levels [26]. Recently, Clement et al. [2] discovered that increases in circulating* Angptl4* reduced proteinuria but at the cost of inducing hypertriglyceridemia. In our analysis p.T266M genotype was not associated with the occurrence of INS disease or clinical severity of the disorder. However, the C allele was more frequent [72 versus 54%] in patients who received IS drug medication instead of GCs only.

Recently, Okamoto et al. [7] identified an association between variants of glypican-5 (*GPC5*) gene and acquired NS (focal segmental glomerulosclerosis, proteinuric IgA-nephropathy) through a genome wide association study and replication analysis. They showed that glypican-5 is localized on podocyte cell surface membranes and that the risk genotype (AA) of the* GPC5* SNP rs16946160 was associated with higher expression. In our study, we observed an association between rs16946160 A allele and early disease onset (16 versus 5%) but we did not find an association between this SNP and INS in general. It is, however, notable that none of our patients and only one of the controls carried the AA genotype. Okamoto et al. found the A allele frequency of controls to be 0.168 and dbSNP (http://www.ncbi.nlm.nih.gov/snp/) puts it at 0.161. In our study, it was only 0.08. Thus, it is possible that due to the frequency differences between populations the association between the risk genotype and INS is not visible in our patients.

Alasehirli et al. [8] found that, in* nNOS* gene polymorphism rs2682826, the TT genotype was associated with INS but not with GC responsiveness. NO attenuates many functions in the kidney and all forms of* NOS* are expressed in the kidney but the role of NO in renal disease is unclear. In our study, we did not find an association of rs2682826 genotypes with INS or with any clinical features of the disease.

Our results of the two cytokines,* IL-13* and* MIF*, whose genetic variants have been associated with NS, were also negative. While Wei et al. [4] reported that 3′UTR SNPs of the* IL-13* gene correlate with long term outcome of INS, we did not see any association between the analyzed SNP and the number of relapses, response to medication, or any other feature.* MIF* is counterregulated by glucocorticoids and the rs755622 SNPs have been studied in association with NS. Vivarelli et al. [6] found that the frequency of C allele was higher in Italian patients than in controls and higher in steroid resistant NS (SRNS) than in steroid sensitive NS (SSNS). Similarly, Berdeli et al. [5] found that GC genotype and C allele were higher in patients than in controls and CC genotype was more frequent in patients with SRNS than in those with SSNS. On the other hand, Choi et al. [22] did not see this association in Korean patients. Similarly, our study did not reveal any association between rs755622 SNP and INS or any of the clinical parameters.

*MDR-1* gene codes for a membranous P-gp, which is a multidrug transporter expressed in the proximal tubule cells. Certain SNPs in* MDR1* gene are believed to affect the expression of the gene or activity of the protein it codes. The common SNP rs1045642 in exon 26 has garnered a lot of attention. It is synonymously variant and it has been suggested that it is not causal itself but linked with another polymorphism or has an effect on DNA structure or RNA stability [12]. Of the other two common SNPs included in this study rs2032582 does lead to amino acid change, Ala899Ser/Thr. This change could possibly increase the drug resistance of the cell [27, 28]. The data on the significance of these SNPs from different studies are contradictory. The distribution of rs2032582 genotypes was found to be significantly different in healthy controls compared to patients in Indian and Egyptian populations [11, 12] while studies in Polish and Korean subjects did not find the association [9, 22].

We did not find any difference in the Finnish patients and controls in rs2032582 genotypes. Wasilewska et al. [9], Jafar et al. [11], and Youssef et al. [12] found an association between rs1045642 and NS (allele T and genotype TT were higher in patients) while Choi et al. [22] did not. All the subjects in these studies come from different populations; thus it is possible that the difference in results is due to genetic heterogeneity among populations. Youssef et al. [12] compared rs1045642 allele frequencies in their Egyptian control subjects and found that the frequencies (C 66.4%, T 33.6%) were consistent with frequencies previously reported in African populations but different from frequencies found in Caucasian, Asian, and Indian populations [11, 29]. In our study, these frequencies were nearly the opposite (C 37.2%, T 62.8%) of those determined by Youssef et al. [12]. This may affect the association between rs1045642 genotypes and NS that was observed in Egyptian population but not in Finnish population. Also, in our study population,* MDR1* SNP rs1045642 CC genotype showed association with higher age of onset (20 versus 0%). Youssef et al. reported similar association for SNPS rs2032582 as well as rs1045642. Other studies did not show this association [9, 22].

In our study, all three* MDR1* SNPs showed association with treatment choices, T allele and TT genotype being more common in patients who needed IS drugs compared to those who were only medicated with GCs, which indicates that T and TT are associated with more complicated form of the disease. Surprisingly, only rs1045642 showed significant association between genotype distribution and GC responsiveness (T allele was more frequent in poor responders), although it must be noted that only ten of our patients were not responsive to GCs; this small cohort size may affect these results.

We carried out haplotype analysis of the three* MDR1* variants. In previous studies, Wasilewska et al. [9] reported significant association of haplotype frequencies with steroid response time. Similar association was observed by Choi et al. [22] and Youssef et al. [12], although, interestingly, the major haplotype linked with this property varies between studies. Our study did not find this association and Cizmarikova et al. [30] reached similar conclusion. The two major haplotypes found in our study were TTT and CGC. These two were predominant in patient samples with combined allele frequency of 70%. The remaining 30% was distributed between eight other haplotypes, none of them reaching 10% frequency. In control patients TTT and CGC were also the most common haplotypes but the distribution was more diverse, as five haplotypes had higher than 10% frequency. Previous studies have also shown that TTT and CGC are prevalent haplotypes both in patients and in control subjects [9, 12, 22, 30]. Interestingly Choi et al. [22] and Youssef et al. [12] found haplotype TGC to have a frequency equal to TTT and CGC and have association to steroid responsiveness while Cizmarikova et al. [30] found the frequency of TGC to be under 3% in patients and in controls and have no association to any clinical attribute. Our results are similar to the latter study as TGC frequency reaches just 10% in control samples and 6.5% in patients. It is possible that the differences are caused by haplotype frequency differences between populations.

*NR3C1* codes for glucocorticoid receptor (GR) that can affect the regulation of many biological functions, including responsiveness to GC, and its functional variability may play a role in the therapeutic response to GC. In this study, we analyzed* NR3C1* SNP rs41423247 and curiously found that patients with more than five relapses carried more frequently heterozygous GC genotype than those with less than five relapses (68 versus 33%). The amount of both CC and GG homozygotes was diminished in these frequent relapsers. The allele distribution between groups with over and under five relapses showed no difference. In some previous studies, G allele (especially as a part of the intron B three-SNP haplotype) has been associated with increased GC sensitivity [31, 32] while others could not confirm the association [14]. It is still unclear how our findings fit in with these studies. Similar increased portion of heterozygous genotype was seen in patients with severe course of the disease compared to those with milder course (63 versus 34%).

An interesting new gene in the context of INS is* GLCCI1*. Tantisira et al. [33] first showed that SNPs rs37973 and rs37972, which are in linkage disequilibrium, associated with poor responsiveness to GCs in asthmatic patients. Soon afterwards, Nishibori et al. [15] showed that Glcci1-protein is highly expressed in glomerular podocytes and its deficiency leads to proteinuria. Based on these findings, Cheong et al. [16] looked to see if these SNPs were playing a role in GC responsiveness in NS but could find no association. Similarly our results show no direct association between the alleles and/or genotypes of either SNP or GC responsiveness. However, while rs37972 showed no significant association with any clinical feature, the frequency of the rs37973 A allele was higher in patients with more than five relapses (70 versus 52%) and in patients who received IS drugs compared to those who received only GC medication (67 versus 50%). To our knowledge this association has not been looked into in other studies.

## 5. Conclusion

The studied genetic variants have little role in the course of NS in Finnish patients. A notable exception to this is MDR1 SNPs whose genotype and allele distribution show significant association to different medication regimes. The genetic background to GC sensitivity is very heterogenic and varies between ethnic groups, which may have to be considered when drawing up treatment strategies for individual patients. More work needs to be done to discover other contributing molecules before the genetics of steroid responsiveness in NS can be understood.

## Figures and Tables

**Table 1:** Primer sequences. *T*_*m*_, annealing temperature.

Gene	SNP	Forward 5′-3′	Reverse 5′-3′	*T*_*m*_ (°C)
*Angptl4*	rs1044250 (c.797C>T)	CTACAAGGCGGGGTTTGGGGAT	AAGTGGAGAAGGGTACGGAGAGGCC	62

*GPC5*	rs16946160 (c.325+1026376G>A)	AGAGTTGACAAGAGTTAAACAGCA	GTCATCTCTGACTCCGCAGTAT	58

*IL-13*	rs848 (c.^**∗**^526C>A)	GTTTGTCACCGTTGGGGATTGG	CAATGTCCCCTCCCCCAGTGTT	62

*MIF*	rs755622 (c.-270G>C)	CTAAGAAAGACCCGAGGCGA	GGCACGTTGGTGTTTACGAT	58

*nNOS*	rs2662826 (c.^**∗**^276C>T)	ACTCCTTGAGTTTTCCTGCTGCGA	CCATGTTCCAGTGGTTTCATGCACCC	62

*MDR1*	rs1128503 (c.1236C>T)	TATCCTGTGTCTGTGAATTGCC	CTGACTCACCACACCAATG	62
	rs2032582 (c.2677G>T/A)	TGCAGGCTATAGGTTCCAGG	TTTAGTTTGACTCACCTTCCCG	60
	rs1045642 (c.3435C>T)	TGTTTTCAGCTGCTTGATGG	AAGGCATGTATGTTGGCCTC	58

*GLCCI1*	rs37972 (c.-1473T>C)	GACCCCTGCATATAGTGCCT	AATGAAACTGAAAGCGTACAAAGA	58
	rs37973 (c.-1106G>A)	AATTCCTTGTTGACCCCTGC	AGCTGAGTTTTCGTGACCAG	62

*NR3C1*	rs41423247 (c.1184+646C>G)	AAATTGAAGCTTAACAATTTTGGC	GCAGTGAACAGTGTACCAGACC	58

**Table 2:** The distribution of observed and expected genotypes. *p* values of 0.05 or above signal consistency with the assumptions of the Hardy-Weinberg Equilibrium.

Gene	SNP	Genotype	Patients				Controls			
			Observed	Expected	*χ* ^2^	*p*	Observed	Expected	*χ* ^2^	*p*
*Angptl4*	rs1044250 (c.797C>T)	CC	35	33.6	0.31	*>0.05*	30	22.3	0.14	*>0.05*
		CT	46	48.7			39	42.0		
		TT	19	17.6			15	19.7		

*GPC5*	rs16946160 (c.325+1026376G>A)	GG	88	88.4	0.41	*>0.05*	86	85.6	0.25	*>0.05*
		GA	12	11.3			14	14.7		
		AA	0	0.4			1	0.6		

*IL13*	rs848 (c.^*∗*^526C>A)	CC	31	33.1	0.71	*>0.05*	45	40.6	3.63	*>0.05*
		CA	53	48.9			38	46.9		
		AA	16	18.1			18	13.6		

*MIF*	rs755622 (c.-270G>C)	GG	61	60.8	0.01	*>0.05*	63	59.5	3.88	*p = 0.05*
		GC	34	34.3			29	36.1		
		CC	5	4.8			9	5.5		

*nNOS*	rs2662826 (c.^*∗*^276C>T)	CC	49	49.0	0.0	*>0.05*	52	48.8	2.38	*>0.05*
		CT	42	42.0			35	41.4		
		TT	9	9.0			12	8.8		

*MDR1*	rs1128503 (c.1236C>T)	CC	22	23.5	0.37	*>0.05*	23	21.1	0.58	*>0.05*
		CT	53	50.0			45	48.8		
		TT	25	26.5			30	28.1		
	rs2032582 (c.2677G>T/A)	GG	17	19.4	1.66	*>0.05*	24	23.5	0.36	*>0.05*
		GT	49	45.8			45	46.5		
		TT	26	27.0			24	23.0		
		TA	3	4.2			2	2.4		
		GA	5	3.5			3	2.4		
		AA	0	0.2			0	0.1		
	rs1045642 (c.3435C>T)	CC	13	14.1	0.21	*>0.05*	10	13.6	2.41	*>0.05*
		CT	49	46.9			53	45.8		
		TT	38	39.1			35	38.6		

*GLCCI1*	rs37972 (c.-1473T>C)	CC	35	35.4	0.03	*>0.05*	25	29.0	2.63	*>0.05*
		CT	49	48.2			56	48.1		
		TT	16	16.4			16	20.0		
	rs37973 (c.-1106G>A)	AA	33	33.6	0.07	*>0.05*	24	27.8	2.60	*>0.05*
		GA	50	48.7			52	44.4		
		GG	17	17.6			14	17.8		

*NR3C1*	rs41423247 (c.1184+646C>G)	GG	32	30.8	0.24	*>0.05*	37	34.2	1.53	*>0.05*
		GC	47	49.4			37	42.6		
		CC	21	19.8			16	13.2		

**Table 3:** Comparison of patient and control genotype distribution. OR, odds ratio. CI, confidence interval. *p* values lower than 0.05 are marked with *∗*.

Gene	SNP	Genotype	Patients (%)	Controls (%)	OR (95% CI)	*p*
			*n* = 100	*n* = 84		
*Angptl4*	rs1044250 (c.797C>T)	CC	35 (35)	30 (36)	Reference	
		CT	46 (46)	39 (46)	1.01 (0.53−1.93)	*0.974*
		TT	19 (19)	15 (18)	1.09 (0.47−2.53)	*0.847*
		C	116 (58)	99 (58.9)	Reference	
		T	84 (42)	69 (41.1)	1.04 (0.69−1.58)	*0.857*

			*n* = 100	*n* = 101		
*GPC5*	rs16946160 (c.325+1026376G>A)	GG	88 (88)	86 (85)	Reference	
		GA	12 (12)	14 (14)	0.84 (0.36−1.92)	*0.674*
		AA	0 (0)	1 (1)	6 × 10^−7^ (0−5.75)	*0.236*
		G	188 (94)	186 (92.1)	Reference	
		A	12 (6)	16 (7.9)	0.74 (0.34−1.60)	*0.449*

			*n* = 100	*n* = 101		
*IL13*	rs848 (c.^**∗**^526C>A)	CC	31 (31)	45 (45)	Reference	
		CA	53 (53)	38 (38)	2.03 (1.10−3.79)	0.024^**∗**^
		AA	16 (16)	18 (18)	1.29 (0.57−2.92)	*0.540*
		C	115 (57.5)	126 (63)	Reference	
		A	85 (42.5)	74 (37)	1.26 (0.84−1.89)	*0.261*

			*n* = 100	*n* = 101		
*MIF*	rs755622 (c.-270G>C)	GG	61 (61)	63 (62)	Reference	
		GC	34 (34)	29 (29)	1.21 (0.66−2.23)	*0.537*
		CC	5 (5)	9 (9)	0.57 (0.17−1.76)	*0.335*
		G	156 (78)	155 (76.7)	Reference	
		C	44 (22)	47 (23.3)	0.93 (0.58−1.49)	*0.761*

			*n* = 100	*n* = 99		
*nNOS*	rs2662826 (c.^**∗**^276C>T)	CC	49 (49)	52 (53)	Reference	
		CT	42 (42)	35 (35)	1.27 (0.70−2.32)	*0.425*
		TT	9 (9)	12 (12)	0.80 (0.30−2.05)	*0.636*
		C	140 (70)	139 (70.2)	Reference	
		T	60 (30)	59 (29.8)	1.01 (0.66−1.55)	*0.965*

			*n* = 100	*n* = 98		
*MDR1*	rs1128503 (c.1236C>T)	CC	22 (22)	23 (23)	Reference	
		CT	53 (53)	45 (46)	1.60 (0.82−3.19)	*0.169*
		TT	25 (25)	30 (31)	1.48 (0.68−3.29)	*0.328*
		C	97 (48.5)	91 (46.4)	Reference	
		T	103 (51.5)	105 (53.6)	0.92 (0.62−1.37)	*0.680*
			*n* = 100	*n* = 98		
	rs2032582 (c.2677G>T/A)	GG	17 (17)	24 (24)	Reference	
		GT	49 (49)	45 (45)	1.54 (0.74−3.26)	*0.253*
		TT	26 (26)	24 (24)	1.53 (0.67−3.55)	*0.316*
		TA	3 (3)	2 (2)	2.12 (0.32−17.42)	*0.432*
		GA	5 (5)	3 (3)	2.35 (0.51−12.75)	*0.274*
		G	88 (44)	96 (49)	Reference	
		T	104 (52)	95 (48.5)	1.19 (0.80−1.79)	*0.386*
		A	8 (4)	5 (2.6)	1.75 (0.56−5.96)	*0.338*
			*n* = 100	*n* = 98		
	rs1045642 (c.3435C>T)	CC	13 (13)	10 (10)	Reference	
		CT	49 (49)	53(54)	0.711 (0.28−1.76)	*0.462*
		TT	38 (38)	35 (36)	0.84 (0.32−2.14)	*0.708*
		C	75 (37.5)	73 (37.2)	Reference	
		T	125 (62.5)	123 (62.8)	0.99 (0.66−1.49)	*0.958*

			*n* = 100	*n* = 97		
*GLCCI1*	rs37972 (c.-1473T>C)	CC	35 (35)	25 (26)	Reference	
		CT	49 (49)	56 (58)	0.63 (0.38−1.18)	*0.149*
		TT	16 (16)	16 (16)	0.71 (0.30−2.70)	*0.444*
		C	119 (59)	106 (54.6)	Reference	
		T	81 (41)	88 (45.4)	0.82 (0.55−1.22)	*0.330*
			*n* = 100	*n* = 90		
	rs37973 (c.-1106G>A)	AA	33 (33)	24 (27)	Reference	
		GA	50 (50)	52 (58)	0.70 (0.36−1.34)	*0.282*
		GG	17 (17)	14 (16)	0.88 (0.37−2.15)	*0.782*
		A	116 (58)	100 (55.6)		
		G	84 (42)	80 (44.4)	0.91 (0.60−1.36)	*0.631*

			*n* = 100	*n* = 90		
*NR3C1*	rs41423247 (c.1184+646C>G)	GG	32 (32)	37 (41)	Reference	
		GC	47 (47)	37 (41)	1.47 (0.78−2.80)	*0.238*
		CC	21 (21)	16 (18)	1.52 (0.68−3.43)	*0.308*
		G	111 (55.5)	111 (61.7)	Reference	
		C	89 (44.5)	69 (38.3)	1.29 (0.86−1.95)	*0.223*

**Table 4:** Comparison of genotype and allele frequencies of functional kidney gene variants and selected clinical features. *p* values lower than 0.05 are marked with *∗*.

Gene	SNP	Genotype	Relapses			Frequent relapser (%)			Age of onset (%)			Response to steroids			Treatment		
			<5	>5	*p*	No	Yes	*p*	<3	>3	*p*	Normal	Slow/NR	*p*	Only GC	IS	*p*
			*n* = 52	*n* = 25		*n* = 41	*n* = 40		*n* = 16	*n* = 65		*n* = 67	*n* = 13		*n* = 42	*n* = 39	
*Angptl4*	rs1044250 (c.797C>T)	CC	20 (38)	11 (44)	0.9426	14 (34)	18 (44)	0.4133	8 (50)	24 (37)	0.3311	25 (37)	6 (46)	0.8494	12 (29)	20 (51)	0.0679
		CT	25 (48)	11 (44)		19 (46)	18 (44)		6 (38)	31 (48)		32 (48)	5 (38)		21 (50)	16 (41)	
		TT	7 (13)	3 (12)		8 (20)	4 (10)		2 (13)	10 (15)		10 (15)	2 (15)		9 (21)	3 (8)	
		C	65 (62.5)	33 (66)	0.7230	47 (57)	54 (68)	0.1977	22 (69)	79 (61)	0.5416	82 (61)	17 (65)	0.8094	45 (54)	56 (72)	0.0228^**∗**^
		T	39 (37.5)	17 (34)		35 (43)	26 (33)		10 (31)	51 (39)		52 (39)	9 (35)		39 (46)	22 (28)	

*GPC5*	rs16946160 (c.325+1026376G>A)	GG	47 (90)	20 (80)	0.2789	37 (90)	33 (83)	0.3493	11 (69)	59 (91)	0.0359^**∗**^	58 (87)	11 (85)	1.000	38 (90)	32 (82)	0.3389
		GA	5 (10)	5 (20)		4 (10)	7 (18)		5 (31)	6 (9)		9 (13)	2 (15)		4 (10)	7 (18)	
		AA	0 (0)	0 (0)		0 (0)	0 (0)		0 (0)	0 (0)		0 (0)	0 (0)		0 (0)	0 (0)	
		G	99 (95)	45 (90)	0.2951	78 (95)	73 (91)	0.3663	27 (84)	124 (95)	0.0421^**∗**^	125 (93)	24 (92)	0.6322	80 (95)	71 (91)	0.3567
		A	5 (5)	5 (10)		4 (5)	7 (9)		5 (16)	6 (5)		9 (7)	2 (8)		4 (5)	7 (9)	

*IL13*	rs848 (c.^**∗**^526C>A)	CC	18 (35)	8 (32)	0.8456	10 (24)	16 (40)	0.3114	7 (44)	19 (29)	0.6420	24 (36)	2 (15)	0.3703	15 (36)	11 (28)	0.7568
		CA	25 (48)	14 (56)		24 (59)	19 (48)		7 (44)	35 (54)		33 (49)	9 (69)		21 (50)	21 (54)	
		AA	9 (17)	3 (12)		7 (17)	5 (13)		2 (13)	11 (17)		10 (15)	2 (15)		6 (14)	7 (18)	
		C	61 (59)	30 (60)	1.000	44 (54)	51 (64)	0.2056	21 (66)	73 (56)	0.4246	81 (60)	13 (50)	1.000	51 (61)	43 (55)	0.5253
		A	43 (41)	20 (40)		38 (46)	29 (36)		11 (34)	57 (44)		53 (40)	13 (50)		33 (39)	35 (45)	

*MIF*	rs755622 (c.-270G>C)	GG	31 (60)	16 (64)	1.000	26 (63)	24 (60)	0.9284	9 (56)	40 (62)	0.0634	40 (60)	8 (62)	1.000	25 (60)	24 (62)	0.7481
		GC	18 (35)	8 (32)		13 (32)	14 (35)		7 (44)	21 (32)		23 (34)	5 (38)		14 (33)	14 (36)	
		CC	3 (6)	1 (4)		2 (5)	2 (5)		0 (0)	4 (6)		4 (6)	0 (0)		3 (7)	1 (3)	
		G	80 (77)	40 (80)	0.8359	65 (79)	62 (78)	0.8497	25 (78)	101 (78)	1.000	103 (77)	21 (81)	1.000	64 (76)	62 (79)	0.7063
		C	24 (23)	10 (20)		17 (21)	18 (23)		7 (22)	29 (22)		31 (23)	5 (19)		20 (24)	16 (21)	

*nNOS*	rs2662826 (c.^**∗**^276C>T)	CC	24 (46)	10 (4)	0.7393	19 (46)	17 (43)	0.2027	5 (31)	31 (48)	0.4080	29 (43)	7 (54)	0.6178	19 (45)	17 (44)	0.5406
		CT	23 (44)	11 (44)		20 (49)	16 (40		8 (50)	28 (43)		31 (46)	4 (31)		20 (48)	16 (41)	
		TT	5 (10)	4 (16)		2 (5)	7 (18)		3 (19)	6 (9)		7 (10)	2 (15)		3 (7)	6 (15)	
		C	71 (68)	31 (62)	0.4702	58 (71)	50 (63)	0.4075	18 (56)	90 (69)	0.2089	89 (66)	18 (69)	0.458	58 (69)	50 (64)	0.5103
		T	33 (32)	19 (38)		24 (29)	30 (38)		14 (44)	40 (31)		45 (34)	8 (31)		26 (31)	28 (36)	

**Table 5:** Comparison of genotype and allele frequencies of glucocorticoid metabolism gene variants and selected clinical features. GC, glucocorticoid medication. NR, no response. IS, immunosuppressive drugs. *p* values lower than 0.05 are marked with *∗*.

Gene	SNP	Genotype	Relapses			Frequent relapser (%)			Age of onset (%)			Response to GC			Treatment		
			<5	>5	*p*	No	Yes	*p*	<3	>3	*p*	Normal	Slow/NR	*p*	Only GCs	IS	*p*
			*n* = 52	*n* = 25		*n* = 41	*n* = 40		*n* = 16	*n* = 65		*n* = 67	*n* = 13		*n* = 42	*n* = 39	
*MDR1*	rs1128503 (c.1236C>T)	CC	12 (23)	4 (16)	0.2330	9 (22)	8 (20)	0.4526	1 (6)	16 (25)	0.2863	14 (21)	3 (23)	1.000	12 (29)	5 (17)	0.01^**∗**^
		CT	29 (56)	11 (44)		23 (56)	18 (45)		10 (63)	31 (48)		34 (51)	6 (46)		24 (57)	7 (24)	
		TT	11 (21)	10 (40)		9 (22)	14 (35)		5 (31)	18 (28)		19 (28)	4 (31)		6 (14)	17 (59)	
		C	53 (51)	19 (38)	0.1678	41 (50)	34 (43)	0.3496	12 (38)	63 (48)	0.3240	62 (46)	12 (46)	0.0566	48 (57)	17 (29)	0.0012^**∗**^
		T	51 (49)	31 (62)		41 (50)	46 (58)		20 (63)	67 (52)		72 (54)	14 (54)		36 (43)	41 (71)	
	rs2032582 (c.2677G>T/A)	GG	9 (17)	4 (16)	0.5655	6 (15)	7 (18.5)	0.3793	1 (6)	12 (18)	0.3311	12 (18)	1 (8)	0.4136	10 (24)	3 (8)	0.0067^**∗**^
		GT	25 (48)	11 (44)		22 (54)	17 (43.5)		7 (44)	32 (49)		32 (48)	6 (46)		22 (53)	17 (44)	
		TT	13 (25)	10 (40)		9 (22)	15 (38.5)		7 (44)	17 (26)		20 (30)	4 (31)		6 (14)	18 (46)	
		TA	2 (4)	0 (0)		2 (5)	0 (0)		1 (6)	1 (2)		1 (1)	1 (8)		2 (5)	0 (0)	
		GA	3 (6)	0 (0)		2 (5)	1 (3.5)		0	3 (5)		2 (3)	1 (8)		2 (5)	1 (3)	
		G	46 (44)	19 (38)	0.1985	36 (44)	32 (40)	0.3448	9 (28)	59 (45)	0.1642	58 (43)	9 (35)	0.0529	44 (52)	24 (31)	0.0028^**∗**^
		T	53 (51)	31 (62)		42 (51)	47 (59)		22 (69)	67 (52)		73 (54)	15 (58)		36 (43)	53 (68)	
		A	5 (6)	0 (0)		4 (5)	1 (1)		1 (3)	4 (3)		3 (3)	2 (8)		4 (5)	1 (1)	
	rs1045642 (c.3435C>T)	CC	9 (17)	4 (16)	1.000	8 (20)	5 (12)	0.4179	0 (0)	13 (20)	0.0315^**∗**^	12 (18)	1 (8)	0.8447	10 (24)	3 (8)	0.0389^**∗**^
		CT	23 (44)	11 (44)		19 (46)	16 (39)		11 (69)	24 (37)		28 (42)	6 (46)		20 (48)	15 (38)	
		TT	20 (38)	10 (40)		14 (34)	19 (46)		5 (31)	28 (43)		27 (40)	6 (46)		12 (29)	21 (54)	
		C	41 (39)	19 (38)	1.000	35 (43)	26 (33)	1.1977	11 (34)	50 (38)	0.8389	52 (39)	8 (31)	0.0113^**∗**^	40 (48)	21 (27)	0.0092^**∗**^
		T	63 (61)	31 (62)		47 (57)	54 (68)		21 (66)	80 (62)		82 (61)	18 (69)		44 (52)	57 (73)	

*GLCCI1*	rs37972 (c.-1473T>C)	CC	16 (31)	11 (44)	0.4931	13 (32)	15 (38)	0.8291	8 (50)	20 (31)	0.2289	24 (36)	4 (31)	1.000	11 (26)	17 (44)	0.2726
		CT	26 (50)	11 (44)		21 (51)	20 (50)		5 (31)	35 (54)		33 (49)	7 (54)		23 (55)	17 (44)	
		TT	10 (19)	3 (12)		7 (17)	5 (13)		3 (19)	10 (15)		10 (15)	2 (15)		8 (19)	5 (13)	
		C	58 (56)	33 (66)	0.2938	47 (57)	50 (63)	0.525	21 (66)	75 (58)	0.5473	81 (60)	15 (58)	1.000	45 (54)	51 (65)	0.1506
		T	46 (44)	17 (34)		35 (43)	30 (38)		11 (34)	55 (42)		53 (40)	11 (42)		39 (46)	27 (35)	
	rs37973 (c.-1106G>A)	AA	14 (27)	12 (48)	0.1197	12 (29)	15 (38)	0.6024	8 (50)	19 (29)	0.1861	22 (33)	5 (38)	0.8423	9 (21)	18 846)	0.0623
		GA	26 (50)	11 (44)		21 (51)	20 (50)		5 (31)	35 (54)		33 (49)	7 (54)		24(57)	16 (41)	
		GG	12 (23)	2 (8)		8 (20)	5 (13		3 (19)	11 (17)		12 (18)	1 (8)		9 (21)	5 (13)	
		A	54 (52)	35 (70)	0.0377^**∗**^	45 (55)	50 (63)	0.3427	21 (66)	73 (56)	0.4246	77 (57)	17 (65)	0.6321	42 (50)	52 (67)	0.0387
		G	50 (48)	15 (30)		37 (45)	30 (38)		11 (34)	57 (44)		57 (43)	9 (35)		42 (50)	26 (33)	

*NR3C1*	rs41423247 (c.1184+646C>G)	GG	20 (38)	5 (20)	0.0173^**∗**^	14 (34)	11 (28)	0.0442^**∗**^	5 (31)	21 (32)	0.5928	23 (34)	2 (15)	0.2363	16 (38)	10 (26)	0.0655
		GC	17 (33)	17 (68)		14 (34)	24 (60)		6 (38)	31 (48)		31 (46)	6 (46)		14 (33)	23 (59)	
		CC	15 (29)	3 (12)		13 (32)	5 (13)		5 (31)	13 (20)		13 (19)	5 (38)		12 (29)	6 (15)	
		G	57 (55)	27 (54)	1.000	42 (51)	46 (58)	0.4352	16 (50)	73 (56)	0.5571	77 (57)	10 (38)	0.3479	46 (55)	43 (55)	1.000
		C	47 (45)	23 (46)		40 (49)	34 (43)		16 (50)	57 (44)		57 (43)	16 (62)		38 (45)	35 (45)	

**Table 6:** Comparison of haplotype distributions of the MDR1 loci in patients and controls as well as in patients with less than 5 relapses and patients with five or more relapses. Variants in order rs1128503, rs2032582, and rs1045642. *p* values lower than 0.05 are marked with *∗*.

Haplotype	Patients (*n* = 82)		Controls (*n* = 98)		OR (95% CI)	*p*
	*N*	%	*N*	%		
CTC	69	42.2	46	23.4	0.42 (0.27–0.66)	**0.002**
TGT	45	27.5	36	18.6	0.60 (0.36–0.98)	**0.0412**
TTC	13	8.1	31	15.6	2.18 (1.10–4.33)	**0.0254**
CGC	7	4.5	25	12.6	3.28 (1.38–7.79)	**0.0072**
TGC	11	6.5	20	10.0	1.58 (0.73–3.40)	*0.242*
CGT	7	4.2	15	7.8	1.86 (0.74–4.67)	*0.1877*
TTT	2	1.3	15	7.7	6.71 (1.51–29.80)	**0.0123**
Other	9	5.7	8	4.3	0.73 (0.28–1.94)	*0.5425*

Haplotype	Relapses <5 (*n* = 52)		Relapses >5 (*n* = 25)		OR (95% CI)	*p*
	*N*	%	*N*	%		

CTC	42	40.6	28	55.3	1.88 (0.95–3.71)	**0.07**
TGT	32	30.4	16	31.4	1.06 (0.51–2.91)	*0.88*
TGC	10	9.5	1	2.4	0.19 (0.02–1.54)	*0.1205*
TTC	6	6.2	2	4.2	0.68 (0.13–3.50)	*0.64*
CGT	2	2.0	2	4.0	2.13 (0.29–15.54)	*0.4578*
CTT	3	3.2	1	2.6	0.69 (0.07–6.78)	*0.7479*
Other	8	8.1	0	0.0	0.11 (0.01–1.99)	*0.1359*

**Table 7:** Comparison between the found associations of the analyzed SNPs in this study and in previous studies.

Gene	Found association	Current study	Referenced study
*GPC5*	*rs16946160 (c.325+1026376G>A)*		
	Association of AA genotype with NS	No	Yes [7]
	Association of A allele with disease onset	Yes	
	A allele frequency	0,168	0,08 [7]

*IL13*	*rs848 (c.* ^**∗**^ *526C>A)*		
	Association of genotype distribution with long term outcome	No	Yes [4]

*MIF*	*rs755622 (c.-270G>C)*		
	Association of C allele and/or GC genotype with NS	No	Yes [5], yes [6], no [22]
	Association of CC genotype with GC resistance	No	Yes [5], yes [6], no [22]

*nNOS*	*rs2662826 (c.* ^**∗**^ *276C>T)*		
	Association of TT genotype with NS	No	Yes [8]
	Association of TT genotype with GC responsiveness	No	No [8]

*MDR1*	*rs1128503 (c.1236C>T)*		
	Association of T allele and/or TT genotypes with IS medication need	Yes	
	*rs2032582 (c.2677G>T/A)*		
	Association of genotype distribution with NS	No	No [9], yes [11], yes [12], no [22]
	Association of CC genotype with age of onset	No	No [9], no [11], yes [12], no [22]
	Association of T allele and/or TT genotype with GC responsiveness	Yes	
	Association of T allele and/or TT genotypes with IS medication need	Yes	
	*rs1045642 (c.3435C>T)*		
	Association of T allele and/or TT genotype with NS	No	Yes [9], yes [11], yes [12], no [22]
	Allele frequencies of controls (C/T, %)	37.2/62.8	45,2/54,8 [9], 66.4/33.6 [11], 58.5/41.5 [12], 42.0/58.0 [22], 42.4/57.6 [30]
	Association of CC genotype with age of onset	Yes	No [9], no [11], yes [12], no [22]
	Association of T allele and/or TT genotypes with IS medication need	Yes	
	*Haplo type *		
	Association with GC responsiveness	No	Yes [9], yes [12], yes [22], no [30]
	Frequency of TGC haplotype (case/control, %)	6.5.2010	1.1^*∗*^ [9], 8.3/6.5 [11], 21,7/18.6 [12], 21.2/18 [22], 1.1/2.3 [30]

*GLCCI1*	*rs37972 (c.-1473T>C)*		
	Association of genotype distribution with GC responsiveness	No	No [16]
	*rs37973 (c.-1106G>A)*		
	Association of genotype distribution with GC responsiveness	No	No [16]
	Association of A allele with patients with more than five relapses	Yes	
	Association of A allele with IS medication need	Yes	

^*∗*^Only control.
